# Case report: biallelic *DNMT3A* mutations in acute myeloid leukemia

**DOI:** 10.3389/fonc.2023.1205220

**Published:** 2023-06-28

**Authors:** Cosimo Cumbo, Paola Orsini, Luisa Anelli, Antonella Zagaria, Maria Federica Iannò, Loris De Cecco, Crescenzio Francesco Minervini, Nicoletta Coccaro, Giuseppina Tota, Elisa Parciante, Maria Rosa Conserva, Immacolata Redavid, Francesco Tarantini, Angela Minervini, Paola Carluccio, Anna De Grassi, Ciro Leonardo Pierri, Giorgina Specchia, Pellegrino Musto, Francesco Albano

**Affiliations:** ^1^ Department of Precision and Regenerative Medicine and Ionian Area (DiMePRe-J), Hematology and Stem Cell Transplantation Unit, University of Bari “Aldo Moro”, Bari, Italy; ^2^ Fondazione IRCCS Istituto Nazionale dei Tumori, Milan, Italy; ^3^ Molecular Mechanisms Unit, Department of Research Fondazione IRCCS Istituto Nazionale dei Tumori, Milan, Italy; ^4^ Laboratory of Biochemistry, Molecular and Computational Biology, Department of Biosciences, Biotechnologies and Biopharmaceutics, University of Bari, Bari, Italy; ^5^ School of Medicine, University of Bari “Aldo Moro”, Bari, Italy

**Keywords:** *DNMT3A*, biallelic mutations, acute myeloid leukemia, hypermethylation, cell differentiation

## Abstract

*DNMT3A* gene mutations, detected in 20-25% of *de novo* acute myeloid leukemia (AML) patients, are typically heterozygous. Biallelic variants are uncommon, affecting ~3% of cases and identifying a worse prognosis. Indeed, two concomitant *DNMT3A* mutations were recently associated with shorter event-free survival and overall survival in AML. We present an AML case bearing an unusual *DNMT3A* molecular status, strongly affecting its function and strangely impacting the global genomic methylation profile. A 56-year-old Caucasian male with a diagnosis of AML not otherwise specified (NOS) presented a complex *DNMT3A* molecular profile consisting of four different somatic variants mapping on different alleles *(in trans*). 3D modelling analysis predicted the effect of the *DNMT3A* mutational status, showing that all the investigated mutations decreased or abolished DNMT3A activity. Although unexpected, DNMT3A’s severe loss of function resulted in a global genomic hypermethylation in genes generally involved in cell differentiation. The mechanisms through which DNMT3A contributes to AML remain elusive. We present a unique AML case bearing multiple biallelic *DNMT3A* variants abolishing its activity and resulting in an unexpected global hypermethylation. The unusual DNMT3A behavior described requires a reflection on its role in AML development and persistence, highlighting the heterogeneity of its deregulation.

## 1 Introduction

DNA methyltransferase (DNMT) proteins are a family of factors responsible for DNA methylation, an epigenetic modification involved in development, aging, and cancer (1). DNMT3A is a *de novo* DNMT with a key role in hematopoiesis, implicated in the balance between the self-renewal and differentiation of hematopoietic stem cells (HSCs) (2). It contains three well-characterized domains: the Pro-Trp-Trp-Pro (PWWP) domain which binds DNA and specific histone marks in gene bodies, the ATRX-DNMT3-DNMT3L (ADD) domain which binds histone 3 (H3), and the highly conserved methyltransferase (MTase) domain which attaches a methyl group to the C5 position of cytosine after binding the S-adenosyl methionine (SAM) cofactor (3).

*DNMT3A* mutations are detected in several hematologic malignancies (often occurring as early events during leukemogenesis) and in 20-25% of *de novo* acute myeloid leukemia (AML) patients (4). In myeloid malignancies, *DNMT3A* variants are typically heterozygous (4); in fact, *DNMT3A* acts as a haploinsufficient tumor suppressor gene in myeloid diseases when cooperating mutations are present (3). In AML, biallelic *DNMT3A* mutations are uncommon, affecting ~3% of patients, as reported in The Cancer Genome Atlas (TCGA) AML (5), and identifying a worse prognosis. Indeed, two concomitant DNMT3A mutations were recently associated with shorter event-free survival and overall survival in AML patients (6). We present an AML case bearing a complex and unusual *DNMT3A* molecular status, strongly affecting the gene function and strangely impacting the global genomic methylation profile.

## 2 Case presentation

A 56-year-old Caucasian male was referred to our center for pretransplant eligibility evaluation. He had previously been diagnosed with AML not otherwise specified (NOS) according to the 2016 World Health Organization (WHO) classification (7). At the time of diagnosis, the karyotype was normal; molecular testing revealed the presence of the *FLT3* internal tandem duplication (ITD) mutation. His past medical history was remarkable for chronic bronchitis, benign prostatic hypertrophy and a Brugada-like ECG pattern. He achieved complete remission (CR) after induction therapy with the 7 + 3 regimen and subsequent consolidation cycles. Given the presence of a fully matched sibling donor, he successfully underwent allogeneic hematopoietic stem cell transplantation (allo-HSCT). Ten months later, bone marrow (BM) aspirate showed 41% of myeloid blasts, mixed chimerism, and molecular testing was positive for the *FLT3*-ITD mutation. The patient started salvage therapy with second-generation FLT3 inhibitor quizartinib. After 3 months, he died due to uncontrolled sepsis.

The study was approved by the local ethics committee of “Azienda Ospedaliero Universitaria Policlinico di Bari”. Informed consent was obtained from the patient before study inclusion, in accordance with the Declaration of Helsinki. Patient’s records/information were anonymized and de-identified before analysis. Consent for publication was obtained from the patient before his enrolment in the present study.

Next-generation sequencing (NGS) analysis with a customized panel encompassing 26 target genes involved in the pathogenesis of myeloid malignancies was performed on BM samples at the time of diagnosis, at CR and disease relapse (DR) (**Additional file 1**), as previously reported (8).

**Figure 1:**
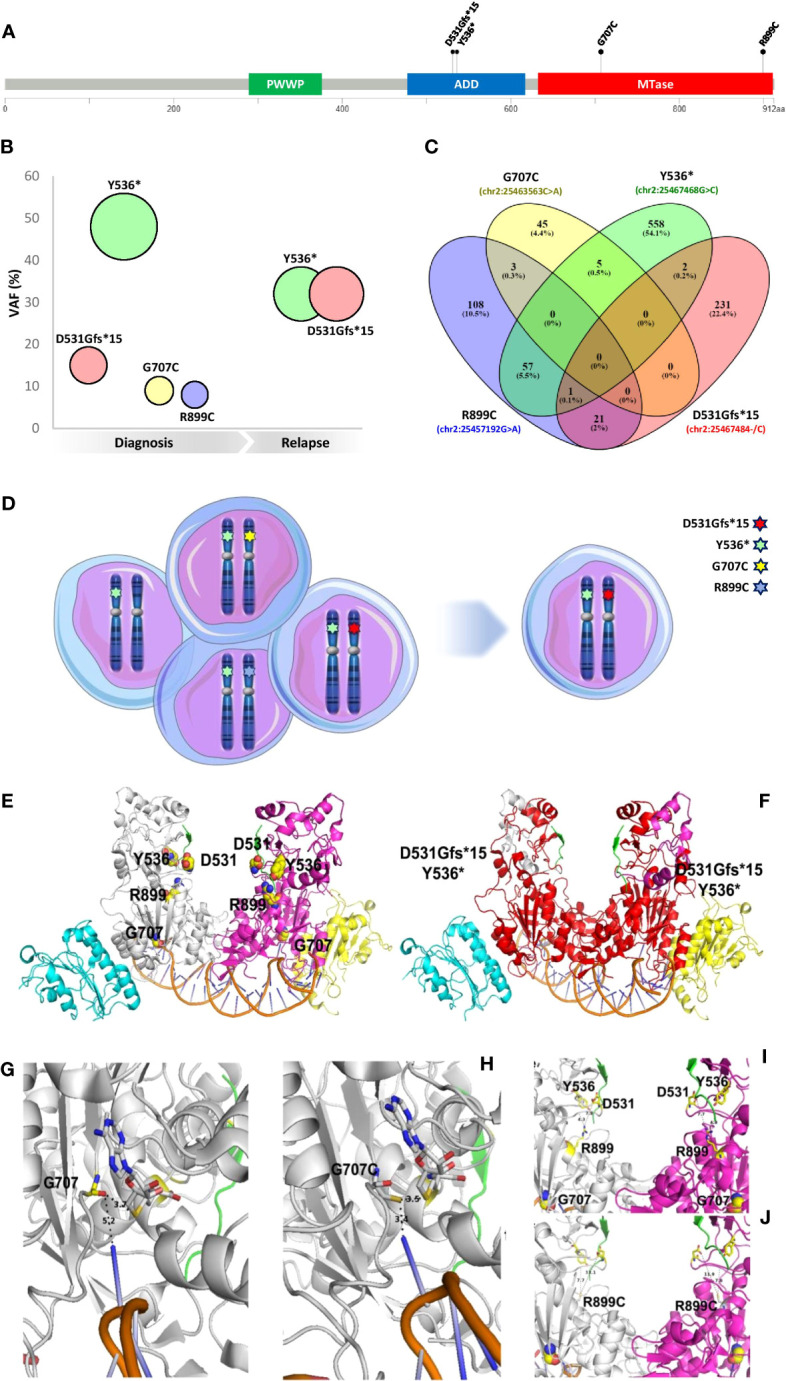
Molecular characterization and 3D modeling of *DNMT3A* mutational profile. **(A)** Map of four variants identified on a linear schematization of the DNMT3A protein (lollipop plots). **(B)** VAF evolution of the mutations detected. **(C)** The Venn diagram of the reads identifiers supporting each variant was retrieved from the corresponding filtered SAM files and visually inspected. **(D)** Clonal configuration from diagnosis (left) to relapse (right). **(E)** Lateral view of the wild type DNMT3A-DNMT3L dimer in a complex with histone H3 (from 4U7T.pdb) and a DNA molecule containing 2 CpG sites (from 5YX2) is reported in the cartoon representation. Histone H3 is depicted in a green cartoon. The DNA molecule is colored orange. DNMT3A monomers are shown in white and magenta. DNMT3L monomers are shown in cyan and yellow. Residues affected by the investigated mutations are reported in yellow spheres and labeled. **(F)** The red cartoon representation indicates the C-terminal domain portions lost from the DNMT3A domains due to the mutations Y536* and D531Gfs*. See the previous panel legend for other color details. **(G, H)** Zoomed view of a DNMT3A C-terminal monomer domain containing the G707 residue [yellow stick, **(G)**] and the corresponding mutation G707C [white stick, **(H)**]. The orange cartoon indicates a groove of the DNA molecule interacting with G707 or G707C. S-adenosyl-homocysteine interacting with G707 or G707C is reported in white sticks. **(I, J)** Zoomed view of a DNMT3A C-terminal dimer domain containing the R899 residue [yellow sticks **(I)**] and the corresponding mutation R899C [white sticks, **(J)**]. Other residues affected by mutations are reported for comparative purposes (see previous panel legends for color details). Dashed lines and labels indicate interactions between protein residues **(G–J)** or between protein residues and nucleotides **(G, H)**.

At diagnosis, NGS analysis identified the presence of a complex *DNMT3A* (NM_022552.4) molecular profile consisting of four different somatic variants: c.1592_1593insG, p.D531Gfs*15 (variant allele frequency (VAF): 15.13%); c.1608C>G, p.Y536* (VAF: 47.87%); c.2119G>T, p.G707C (VAF: 9.04%) and c.2695C>T, p.R899C (VAF: 7.65%) (**Figure 1**). In CR no *DNMT3A* gene variants were identified and at the DR only p.D531Gfs*15 (VAF: 32.75%) and p.Y536* (VAF: 32.12%) were detected (**Figure 1**).

While G707C and R899C are two rare missense pathogenic variants described in hematologic malignancies, D531Gfs*15 and Y536* are novel *DNMT3A* truncating variants never before reported in cancer. We further investigated this unique complex mutational profile to explore the possible role of *DNMT3A* in AML pathogenesis.

A third-generation long-read sequencing (LRS) approach (9, 10) (**Additional file 1**) was performed as reported to phase all variants detected (11, 12). Filtering and comparing the reads containing each of the four *DNMT3A* variants, almost all the reads had one of them, with a small fraction of reads supporting more than one mutation (**Figure 1**), suggesting that all the *DNMT3A* mutations were located on different alleles *(in trans*). Furthermore, based on the VAF value, we established the clonal configuration at diagnosis, mapping the Y536* variant (VAF~50%) on one *DNMT3A* allele of all leukemic cells and the other mutations (with a lower VAF) on the other allele of three different subclones. The identified subclone carrying the two novel truncating variants (Y536* and D531Gfs*15) was responsible for DR (**Figure 1**).

Truncating mutations can induce the nonsense-mediated mRNA decay mechanism (13). To rule out the occurrence of this process, the presence of transcripts carrying the above mutations was verified by direct sequencing (**Additional file** and **Figure 1**), showing that the two truncating variants identified did not cause nonsense-mediated mRNA decay.

A 3D modeling analysis (**Figures 1E**) was performed to predict the effect of the *DNMT3A* mutational status (**Additional file 1**). It was possible to highlight putative DNMT3A orthologs in 6 out of the 14 eukaryotic model organisms, namely in *D. rerio* and *S. formosus* from fishes, *X. laevis* and *X. tropicalis* from amphibians, and *M. musculus* and *H. sapiens* from mammalians. Nevertheless, it was possible to observe that the investigated mutations affect residues that are highly conserved in the DNMT3A C-terminal domain (**Additional** **Figure 2**). Residues 476-912 of this domain appear to be crucial for DNMT3A methylation activity due to their participation in the ADD-catalytic domain (14). Notably, DNMT3A-DNA binding interactions are mediated by specific residues of a loop containing the target recognition domain (TRD, residues R831-F848), the catalytic loop (residues G707-K721) and the homodimeric interface of DNMT3A, together forming a continuous DNA-binding surface (15).

**Figure 2:**
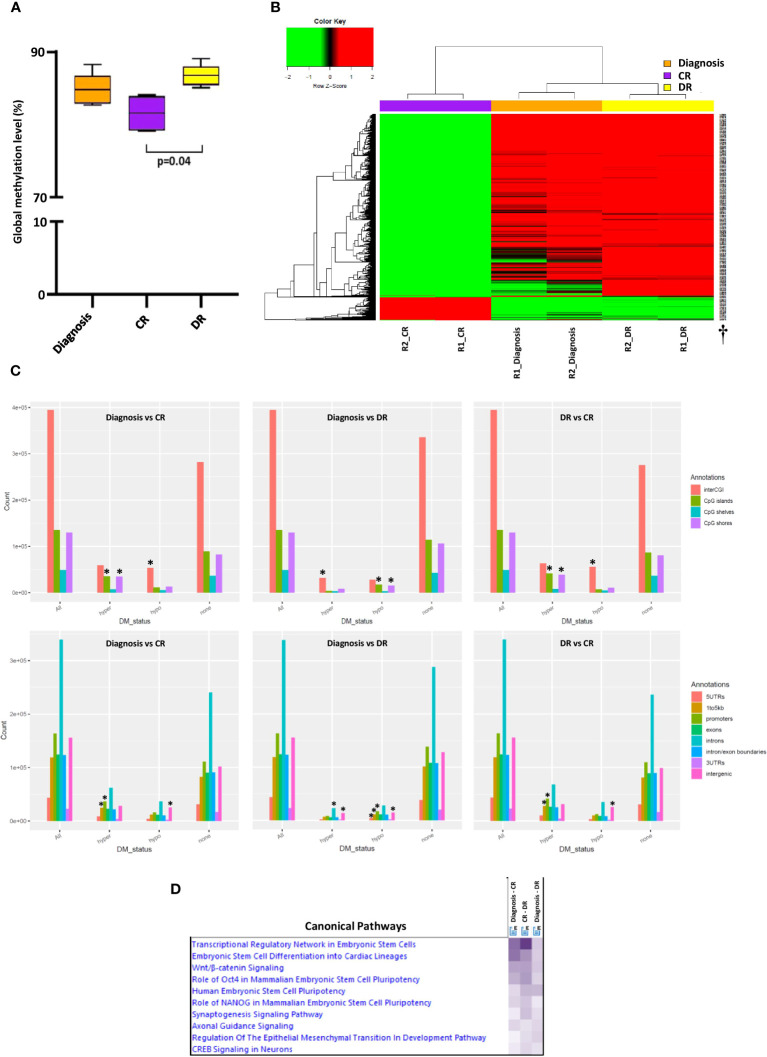
DNA methylation profiling and functional analysis. **(A)** Global methylation status evaluation by droplet digital PCR quantification of the Alu methylation pattern. CR: complete remission, DR: disease relapse. **(B)** Unsupervised hierarchical clustering of the 25000 most variably methylated probes according to standard deviation, applying the Euclidean distance as a distance metric. The heatmap scale shows the range of methylation values, from 0 (unmethylated, green) to 1 (hypermethylated, red). †Probes names. **(C)** Distribution of differentially methylated CpGs (dmCpGs) relative to CGIs and relative distribution of dmCpGs across different genomic regions in the three comparisons. *Statistically differential enrichment (FDR<0.05) of a specific class of CpGs. **(D)** Heatmap of Ingenuity Pathway Analysis (IPA) comparative functional analysis of canonical pathways for the three comparisons performed. The violet intensity is proportional to the differences highlighted in each comparison.

A severe truncation of DNMT3A is caused by variants coding for D531Gfs*15 and Y536*. The truncated proteins lose most of the C-terminal domain (residues 546 to 912 and 536 to 912, respectively), containing both the ADD and the catalytic domain, making the DNMT3A structure unstable, challenging every interaction with DNA molecules and causing loss of the gene activity (**Figure 1**).

The variant producing the missense mutation R899C causes the loss of specific interactions between R899 and residues of the loop containing D531 and Y536 participating in the ADD domain (residues 476-626), and deeply involved in interactions with histone H3 (14), due to the shorter, not charged side chain of the cysteine residue replacing the native arginine (**Figures 1I**).

The variant producing the missense mutation G707C causes an alteration of binding interactions between the DNMT3A catalytic loop (residues G707-K721), containing the G707 residue, the SAM that will provide the methyl group and the DNA double helix that should be methylated (**Figures 1G**). Notably, the replacement of G707 with a cysteine residue may even favour the formation of disulfide bonds with SAM in the catalytic methylation site, altering or reducing/inhibiting DNA-methylation. All the investigated mutations affect DNMT3A function, decreasing or abolishing its activity.

The global DNA methylation profile was evaluated using a droplet digital PCR (ddPCR)-based strategy, quantifying the Alu methylation level to investigate the possible effect of *DNMT3A* loss of function (**Additional file 1**) (16). Although unexpected, DNMT3A’s severe loss of function at diagnosis and DR, results in a global genomic hypermethylation, as compared to CR and a statistically significant difference was observed between CR and DR (81.6% vs 86.7%, respectively, p=0.04) (**Figure 2**).

To better characterize the overall methylation status of the BM samples in the three AML phases (diagnosis, CR, and DR), a genome-wide methylation analysis was performed using the Illumina Methylation EPIC BeadChip microarray (**Additional file 1**), which interrogates over 850,000 CpG sites (CpGs) across the human genome. The multidimensional scaling (MDS) plot revealed a high similarity between technical replicates; moreover, the three samples corresponding to the three phases of disease were further apart in the plot (**Additional Figure 3**). Similarly, unsupervised hierarchical clustering of the most 25000 variably methylated probes (**Additional file 1**) showed that the samples were segregated into three distinct clusters, with diagnosis and DR clustering together and showing a global genomic hypermethylation, as compared to CR (**Figure 2**).

Overall, from the 707818 filtered CpGs, differential methylation analysis revealed that the diagnosis and DR samples had larger numbers of differentially methylated CpG sites (FDR<0.05) as compared to the CR sample (**Additional Table 1**). A significant enrichment (over-representation) of CpG islands (CPGis) and shores (ie., regions up to 2 kb from CpGis) among the hypermethylated CpG sites in the diagnosis and DR samples was observed as compared to the CR sample (**Figure 2**). The overall distribution of hypermethylated CpGs at the gene mapping was suggestive of a significant enrichment of promoters and 1-5kb regions upstream of the TSS in the diagnosis and DR samples as compared to the CR sample; in details, the enrichment of hypermethylated CpGs in these genomic regions was more evident in DR sample compared to diagnosis (**Figure 2**, **Additional Table 2**).

The differentially methylated CpGs were combined for each comparison, identifying 30549, 15563 and 32576 differentially methylated regions (DMRs), respectively. The top 20 DMRs for each comparison are shown in **Additional Table 3**; the top DMRs identified in the diagnosis and DR samples were almost the same.

Furthermore, a global hypermethylation is still evident when the diagnosis and DR samples were compared to U937 cells transduced with wild-type *DNMT3A* (more evident at the DR); on the contrary, comparing our samples to U937 cells transduced with *DNMT3A*-R882C/H, the methylation patterns were almost overlapping (**Additional Table 1**).

Functional analysis with Ingenuity Pathway Analysis (IPA) was also performed on the first 600 genes included in the top DMRs resulting from each comparison, to identify the biological relevance of the DMRs, and then a comparative functional analysis of the three comparisons was performed (**Figure 2**). IPA comparison analysis across the different samples revealed that the “transcriptional regulatory network in embryonic stem cells” canonical pathway was enriched in the DMRs common to the diagnosis and DR samples. Moreover, by comparing the top networks from the three comparisons, significant biological networks related to embryonic development were found to be predominant in the diagnosis and DR samples compared to the CR sample (**Additional Table 4**). This confirms the role of *DNMT3A* in establishing DNA methylation patterns in embryogenesis and suggests a similar effect in AML, defining the HSCs fate (3).

## 3 Discussion

Although widely studied, the mechanisms through which DNMT3A contributes to AML remain elusive. In the case reported here, the atypical *DNMT3A* aberrations and their impact on the global genomic methylation profile offer several points for reflection.

As stated above, *DNMT3A* acts as a haploinsufficient tumor suppressor gene in myeloid malignancies when cooperating mutations are present (3); on the contrary, in T-cell acute lymphoblastic leukemia (T-ALL), a high frequency of non-R882 biallelic mutations has been reported (17), suggesting different selective pressures on *DNMT3A* mutations in myeloid and lymphoid disorders (3). Despite this, the occurrence of multiple *DNMT3A* mutations in AML is recently arousing interest, and the presence of two concomitant *DNMT3A* variants was recently associated with a worse prognosis in AML (6).

We describe a unique AML case bearing four *DNMT3A* variants mapping on different alleles (*in trans*) at diagnosis, decreasing or completely abolishing the gene activity in leukemic cells. Intriguingly, in the clone responsible for DR, carrying two severe truncating mutations, DNMT3A activity was entirely lost.

Although unexpectedly, severe loss of DNMT3A function results in global genomic hypermethylation with a significant enrichment of promoters and 1-5kb regions upstream of the TSS of genes generally involved in cell differentiation in *DNMT3A*-null HSCs (2). Intriguingly, in murine models, the loss of DNMT3A leads to disturbed methylation patterns, shown to be distinct in lymphoid and myeloid diseases, suggesting lineage-specific methylation aberrations; in particular, hypermethylation, mainly at promoter regions, was observed in T-ALL (18). In our case, the mechanism leading to increased methylation is enigmatic; it could originate partly from DNMT3B activity, although its expression level is unchanged in *DNMT3A*-null HSCs (2, 4).

The unusual DNMT3A behavior described in the AML context suggests further reflection on the opportunity to develop targeted therapies for patients bearing this gene mutation. *DNMT3A* R882 and non-R882 patients are considered distinct entities with different genomic and clinical features (19, 20). Furthermore, the mutant burden and the double mutants impact the disease prognosis (6). In other words, in AML, distinct *DNMT3A* molecular profiles seem to differently affect the disease outcome. More studies are warranted to elucidate the *DNMT3A* gene mutation role in AML development and persistence, highlighting the heterogeneity of its deregulation.

## Data availability statement

The datasets presented in this study can be found in online repositories. The names of the repository/repositories and accession number(s) can be found below: https://www.ncbi.nlm.nih.gov/PRJNA836270. https://www.ncbi.nlm.nih.gov/geo/, GSE202488.

## Ethics statement

The studies involving human participants were reviewed and approved by Local ethics committee of “Azienda Ospedaliero Universitaria Policlinico di Bari”. The patients/participants provided their written informed consent to participate in this study.

## Author contributions

Conception and design of the study: CC, PO and FA. Sequencing and ddPCR analyses: CC. 3D modelling analysis: ADG and CLP. Methylation array hybridization: MFI and LDC. Bioinformatic analysis: PO. Acquisition of data and/or analysis and interpretation of data: CC, PO, LA, AZ, CFM, NC, GT, EP, MRC, IR, AM, GS, PM and FA. Clinical data providing: FT and PC. Drafting of the manuscript: FA. All authors contributed to the article and approved the submitted version.
