# Supplementary material for: Case report: biallelic DNMT3A mutations in acute myeloid leukemia
Source: Front Oncol. 2023 Jun 28;13:1205220. doi: 10.3389/fonc.2023.1205220 (PMC10336536; doi:10.3389/fonc.2023.1205220)
Supplement: Supplementary file 1 [file DataSheet_1.pdf]

# *Supplementary Material*

# Biallelic DNMT3A mutations in acute myeloid leukemia

**Cosimo Cumbo, Paola Orsini, Luisa Anelli, Antonella Zagaria, Maria Federica Iannò, Loris De Cecco, Crescenzio Francesco Minervini, Nicoletta Coccaro, Giuseppina Tota, Elisa Parciante, Maria Rosa Conserva, Immacolata Redavid, Francesco Tarantini, Angela Minervini, Paola Carluccio, Anna De Grassi, Ciro Leonardo Pierri, Giorgina Specchia, Pellegrino Musto, Francesco Albano***

*** Correspondence:** Francesco Albano, francesco.albano@uniba.it

## 1 Supplementary Data

### 1.1 Sequencing analyses

*1.1.1. Nucleic acids extraction*

Bone marrow (BM) mononuclear cells were separated by Ficoll density centrifugation. Genomic DNA (gDNA) and total RNA used in all molecular analyses were extracted using the QIAamp DNA Blood Mini Kit and RNeasy Mini Kit (Qiagen, Hilden, Germany), respectively, and quantified by a Qubit 2.0 Fluorometer (Thermo Fisher Scientific, Waltham, MA). One µg of total RNA was reverse transcribed in complementary DNA (cDNA) using the QuantiTect reverse transcription kit (Qiagen, Hilden, Germany).

*1.1.2. Targeted Next Generation Sequencing (NGS)*

For Ion Torrent sequencing (Thermo Fisher Scientific, Waltham, MA), a customized panel, encompassing the full coding regions or specific exons of 26 target genes involved in the pathogenesis of myeloid malignancies, was used; library preparation and data analysis were performed as previously reported (1).

1.1.3. *Long-read sequencing (LRS)*

In order to phase all variants detected, the entire region spanning from exon 14 to exon 23 of the *DNMT3A* gene was amplified in a single amplicon of 10536 bp and prepared for nanopore sequencing (NS). A long-PCR was performed using the PrimeSTAR GXL DNA polymerase (Takara Bio Inc.), a specific primer pair (DNMT3A_F: tgtttgtttgtttaactttgtgtcgct and DNMT3A_R: gggtcatgtcttcagggcttag), 200 ng of gDNA, in a final volume of 50 µl. Thermal-cycling conditions were 98 °C for 10 s, 60 °C for 15 s, 68 °C for 10 min (30 cycles) and 4 °C hold. The PCR product, visualized on an agarose gel (1%), were purified using the QIAquick PCR Purification Kit (Qiagen) and quantified (Nanodrop, Thermo Fisher Scientific). In accordance with the Ligation Sequencing Kit 1D (SQK-LSK108) protocol, the amplicons were prepared for sequencing. After the Platform QC run and priming of the flowcell, the sequencing mix was loaded, and the NC_48Hr_sequencing_FLO-MIN107_SQK-LSK108 protocol was run (MinIONflowcell: FLO-MIN107) on a MinION device (Oxford Nanopore Technologies).
Basecalling of the resulting fast5 files was performed with Guppy toolkit, NanoPlot tool (2) was employed to evaluate nanopore data quality. Reads were aligned on the GRCh37 human reference

genome with minimap2 alignment tool (3) using specific nanopore platform parameters. For phasing analysis, only the reads with a mean read quality greater than 8 and covering the whole genomic interval between the *DNMT3A* variants identified were considered. Phasing analysis was performed as previously described (4), as well as the comparison of the identifiers of the reads supporting each *DNMT3A* mutation. The resulting filtered bam files were visualized on the IGV software (5).
NS data from this study have been submitted to the National Center for Biotechnology Information (NCBI) Short Read Archive (https://www.ncbi.nlm.nih.gov/sra/) under accession Number PRJNA836270.

#### *1.1.4. Direct sequencing*

To rule out the occurrence of nonsense-mediated mRNA decay, the cDNA region of interest was amplified by Polymerase Chain Reaction (PCR) using Platinum Taq DNA Polymerase (Invitrogen, Carlsbad, CA, USA), with 200 ng of gDNA (or cDNA), a specific primer pair (exon17_F: gtccccgacgtacatgatcttc and exon14_R: tctggagtgtgcgtaccagta), in a final volume of 50 uL. Thermal cycling conditions: an initial denaturation of 95 °C for 3 min, 95 °C for 30 s, 60 °C for 30 s, 72 °C for 30 s (35 cycles), 72 °C for 5 min and 4 °C hold. The PCR products were visualized on a 2% agarose gel, purified using the QIAquick PCR Purification Kit (Qiagen, Hilden, Germany), quantified with a Qubit 2.0 fluorometer (Thermo Fisher Scientific, Waltham, MA), and prepared for direct sequencing using the BidDye Terminator v1.1 Cycle Sequencing Kit and a SeqStudio Genetic Analyzer (Thermo Fisher Scientific, Waltham, MA). Electropherograms were then analyzed by visual inspection with the FinchTV software (v.1.4.0; Informer Technologies, Inc.).

### 1.2. 3D modelling analysis

The human protein sequence encoded by the *DNMT3A* gene was queried against the non-redundant reference RNA sequence database using the tblastn version implemented in the NCBI website (https://blast.ncbi.nlm.nih.gov/). For sequence similarity search, 14 eukaryotic model organisms were selected, i.e. two fungi species (*S. cerevisiae* and *S. pombe*), two plant species (*A. thaliana* and *Z. mays*), two nematode species (*C. elegans* and *C. briggsae*), two insect species (*D. melanogaster* and *A. gambiae*), two fish species (*D. rerio* and *S. formosus*), two amphibian species (*X. laevis* and *X. tropicalis*), and two mammalian species (*M. musculus* and *H. sapiens*). Results were filtered for e-value (≤10−40) and coverage (≥80%), and only one isoform per gene, i.e. the longest, was retained for subsequent analysis. Finally, ClustalW was employed for multiple homologous sequence alignment.
The molecular editor PyMOL was used for investigating the 3D structure of the available crystal structures of the C-terminal domain of DNMT3A-DNMT3L in complex with DNA containing 2 CpG sites (6) (PDB_ID: 5YX2) or in complex with histone H3 (7) (PDB_ID: 4U7T) and for generating the four investigated mutants (R899C; G707C; Y536*; D531Gfs*15), starting from the above cited structures (4U7T and 5YX2), in accordance with our validated protocols (8).

### 1.3. DNA methylation analyses

#### *1.3.1. Droplet Digital PCR (ddPCR) assay*

To evaluate the global DNA methylation status, a ddPCR assay for the quantification of Alu methylation pattern was performed (9). Alu repeats, belonging to the Short Interspersed Repetitive Elements (SINEs) class, contain about 25% of CpG sites in the human genome. Alu sequences reside mainly in gene-rich regions and are therefore suitable for evaluating the global DNA methylation

status (10). For each sample a total of 500 ng of gDNA input was prepared (digestion/ligation) for ddPCR EvaGreen assay and 5 pg of the digested/ligated gDNA were used in each ddPCR reaction, as previously reported (9). Each evaluation was replicated five times and the mean value considered to calculate the percentage of methylated consensus Alu sequences for each sample. The replicates performed on diagnosis, remission and relapse samples were compared using the ANOVA test. A *p*-value <0.05 was considered significant.

*1.3.2. Methylation array hybridization*

A more detailed genome-wide methylation analysis was conducted using the Illumina Infinium MethylationEPIC BeadChip arrays, which simultaneously quantify approximately 4% of all CpG dinucleotides. Genomic DNA (500 ng) was bisulfate converted using the EZ DNA Methylation Kit (ZymoResearch, Irvine, CA, USA) according to the manufacturer's recommendations for the Illumina Infinium Assay. Bisulfite-treated samples were processed using the EPIC array (Illumina, San Diego, CA, USA), following manufacturer's instructions. Two technical replicates of diagnosis, remission and relapse samples were included in the MetylationEPIC assay. Microarrays were scanned using an IScan scanner (Illumina). Primary data were acquired by Genome Studio V2011.1 and Methylation Module 1.9.0. The resulting data have been deposited in the Gene Expression Omnibus (GEO) database under the accession number GSE202488.

Methylation data analysis was performed by R4.0.3 using missMethyl, minfi, limma and DMRcate packages (11–14). In brief, from the raw IDAT files sample-specific quality control was performed by plotQC function in the minfi package, confirming that all samples and replicates were of good quality (data not shown). IlluminaHumanMethylationEPIC hg19 annotation files were used for mapping to the human genome. PreprocessFunnorm function (15) was employed for between-array data normalization. Among all 865,859 CpG probes, 1186 probes failed in one or more samples based on detection p-value (p>0.01) were filtered out, as well as 30016 probes with SNPs at CpG site and 126,839 cross-reactive probes, mapping to multiple places in the genome (16,17). Totally, 707818 CpG probes were retained for subsequent analysis. For each CpG probe, M-values and beta values were calculated (18). After normalization and filtering, the relationship between the samples was evaluated by MDS plot (Additional figure 3). Beta values of Cpg probes were sorted according to standard deviation and unsupervised hierarchical clustering analysis of the samples and replicates was performed with the 25000 most variably methylated probes, as established by standard deviation, applying the Euclidean distance as distance metric (Figure 2B).

For pairwise CpG differential methylation analysis, a robust moderated t-test implemented in the R/Bioconductor package limma was used. Resulting p-values were adjusted for multiple comparisons by controlling the False Discovery Rate (FDR) using the Benjamini-Hochberg method; a FDR cut-off of 0.05 was considered for subsequent analyses.

GenomicFeatures and Annotatr (19) R packages were used to annotate all the 707818 filtered CpG sites on the genomic location with respect to CpG and genic annotations. Briefly, CpG shores are defined as 2 kb regions flanking CpG islands, and CpG shelves as the 2 kb regions flanking the respective upstream and downstream shores. Probes not belonging to any of these regions are defined as non-CpG island or open sea. Each probe was assigned to only one category. Genic annotations included 1-5Kb upstream of the transcription start site (TSS), the promoter (< 1Kb upstream of the TSS), 5'UTR, exons, introns, exon/intron boundaries, 3'UTR, and intergenic regions. For genic annotation, if a probe was annotated to multiple exons, it was counted once toward the exons, but if it was annotated to multiple regions for different transcripts (for example an exon and an intron), it would be counted towards both.

A 2 × 2 contingency table was performed for each class of annotated probes in order to study the association between the given subset and the different CGI categories and the genic location. Fisher exact test with alternative “greater” was used to look for enrichment of specific classes of CpGs in hyper- and hypomethylated CpGs compared to the null hypothesis (no enrichment). A significance level of 0.05 was considered for all tests. The odds ratio (OR) for each of the individual tests was used to estimate the enrichment . Odds ratio (OR) and adjusted p-values of Fisher exact tests of all CpGs categories for the three comparisons are reported in Additional table 2.
Annotatr R package was also used to visualize the distribution of differentially methylated CpGs relative to CGIs and across different genomic regions (Figure 2C). From DMPs, region-based analysis to identify differentially methylated regions (DMRs) associated with the three different conditions was performed with DMRcate R package (11).
Methylation profiling of diagnosis and DR samples was also compared to *in silico* methylation data of U937 cells with overexpression of wild-type *DNMT3A*, *DNMT3A* mutant R882C or *DNMT3A* mutant R882H using the Illumina MethylationEpic array (GSE90933).

### 1.4. Functional Analysis

For each comparison, from the differentially methylated regions ranked by Fisher’s multiple comparison statistic, the first 600 genes were retrieved and functional analysis was performed with Ingenuity Pathway Analysis (IPA) (IPA, http://www.ingenuity.com). In order to evaluate the enriched pathways between the three analyses, the IPA comparison analysis function was used, and heatmap was evaluated to visually find trends and similarities across the three comparisons; log p-value was used as statistical measure of similarities across analyses (Figure 2D).

### 1.5. References


1. Cumbo C, Tota G, De Grassi A, Anelli L, Zagaria A, Coccaro N, Tarantini F, Minervini CF, Parciante E, Impera L, et al. RUNX1 gene alterations characterized by allelic preference in adult acute myeloid leukemia. *Leuk Lymphoma* (2021) **0**:1–9. doi:10.1080/10428194.2021.1929960
2. De Coster W, D’Hert S, Schultz DT, Cruts M, Van Broeckhoven C. NanoPack: Visualizing and processing long-read sequencing data. *Bioinformatics* (2018) **34**:2666–2669. doi:10.1093/bioinformatics/bty149
3. Li H. Minimap2: Pairwise alignment for nucleotide sequences. *Bioinformatics* (2018) **34**:3094–3100. doi:10.1093/bioinformatics/bty191
4. Cumbo C, Orsini P, Anelli L, Zagaria A, Minervini CF, Coccaro N, Tota G, Impera L, Parciante E, Conserva MR, et al. Nanopore sequencing sheds a light on the FLT3 gene mutations complexity in acute promyelocytic leukemia. *Leuk Lymphoma* (2021) **62**:1219–1225. doi:10.1080/10428194.2020.1856838
5. Robinson JT, Thorvaldsdóttir H, Winckler W, Guttman M, Lander ES, Getz G, Mesirov JP. Integrative Genome Viewer. *Nat Biotechnol* (2011) **29**:24–6. doi:10.1038/nbt.1754.Integrative
6. Zhang ZM, Lu R, Wang P, Yu Y, Chen D, Gao L, Liu S, Ji D, Rothbart SB, Wang Y, et al. Structural basis for DNMT3A-mediated de novo DNA methylation. *Nat 2018 5547692* (2018) **554**:387–391. doi:10.1038/nature25477
7. Guo X, Wang L, Li J, Ding Z, Xiao J, Yin X, He S, Shi P, Dong L, Li G, et al. Structural insight into autoinhibition and histone H3-induced activation of DNMT3A. *Nat 2014 5177536* (2014) **517**:640–644. doi:10.1038/nature13899

8. Pierri CL, Parisi G, Porcelli V. Computational approaches for protein function prediction: A combined strategy from multiple sequence alignment to molecular docking-based virtual screening. *Biochim Biophys Acta - Proteins Proteomics* (2010) **1804**:1695–1712. doi:10.1016/j.bbapap.2010.04.008
9. Orsini P, Impera L, Parciante E, Cumbo C, Minervini CF, Minervini A, Zagaria A, Anelli L, Coccaro N, Casieri P, et al. Droplet digital PCR for the quantification of Alu methylation status in hematological malignancies. *Diagn Pathol* (2018) **13**:1–11. doi:10.1186/s13000-018-0777-x
10. Lisanti S, Omar WAW, Tomaszewski B, De Prins S, Jacobs G, Koppen G, Mathers JC, Langie SAS. Comparison of methods for quantification of global DNA methylation in human cells and tissues. *PLoS One* (2013) **8**: doi:10.1371/journal.pone.0079044
11. Peters TJ, Buckley MJ, Chen Y, Smyth GK, Goodnow CC, Clark SJ. Calling differentially methylated regions from whole genome bisulphite sequencing with DMRcate. *Nucleic Acids Res* (2021) **49**: doi:10.1093/nar/gkab637
12. Ritchie ME, Phipson B, Wu D, Hu Y, Law CW, Shi W, Smyth GK. Limma powers differential expression analyses for RNA-sequencing and microarray studies. *Nucleic Acids Res* (2015) **43**:e47. doi:10.1093/nar/gkv007
13. Aryee MJ, Jaffe AE, Corrada-Bravo H, Ladd-Acosta C, Feinberg AP, Hansen KD, Irizarry RA. Minfi: A flexible and comprehensive Bioconductor package for the analysis of Infinium DNA methylation microarrays. *Bioinformatics* (2014) **30**:1363–1369. doi:10.1093/bioinformatics/btu049
14. Phipson B, Maksimovic J, Oshlack A. MissMethyl: An R package for analyzing data from Illumina's HumanMethylation450 platform. *Bioinformatics* (2016) **32**:286–288. doi:10.1093/bioinformatics/btv560
15. Fortin JP, Labbe A, Lemire M, Zanke BW, Hudson TJ, Fertig EJ, Greenwood CMT, Hansen KD. Functional normalization of 450k methylation array data improves replication in large cancer studies. *Genome Biol* (2014) **15**:1–17. doi:10.1186/S13059-014-0503-2/FIGURES/10
16. Chen YA, Lemire M, Choufani S, Butcher DT, Grafodatskaya D, Zanke BW, Gallinger S, Hudson TJ, Weksberg R. Discovery of cross-reactive probes and polymorphic CpGs in the Illumina Infinium HumanMethylation450 microarray. *Epigenetics* (2013) **8**:203–209. doi:10.4161/epi.23470
17. Pidsley R, Zotenko E, Peters TJ, Lawrence MG, Risbridger GP, Molloy P, Van Djik S, Muhlhausler B, Stirzaker C, Clark SJ. Critical evaluation of the Illumina MethylationEPIC BeadChip microarray for whole-genome DNA methylation profiling. *Genome Biol* (2016) **17**:1–17. doi:10.1186/s13059-016-1066-1
18. Du P, Zhang X, Huang CC, Jafari N, Kibbe WA, Hou L, Lin SM. Comparison of Beta-value and M-value methods for quantifying methylation levels by microarray analysis. *BMC Bioinformatics* (2010) **11**: doi:10.1186/1471-2105-11-587
19. Cavalcante RG, Sartor MA. Annotatr: Genomic regions in context. *Bioinformatics* (2017) **33**:2381–2383. doi:10.1093/bioinformatics/btx183

## 2 Supplementary Figures and Tables

**File format:** .xls
**Title of data:** Additional table 1
**Description of data:** Number of differential methylated CpG sites for multiple comparisons. (FDR < 0.05).

**File format:** .xls
**Title of data:** Additional table 2
**Description of data:** Enrichment analysis results for all the three comparisons compared to the null distribution of CpG probes. For each class of CpGs, the Odds Ratio (OR) and adjusted p-values of Fisher exact tests are shown.

**File format:** .xls
**Title of data:** Additional table 3
**Description of data:** Top 20 differentially methylated regions (DMRs) resulting for each comparison from region-based analysis with the DMRcate R package.

**File format:** .xls
**Title of data:** Additional table 4
**Description of data:** Top networks list resulting from the Ingenuity Pathway Analysis (IPA) for each comparison, with the specific scores associated with each network.

**File format:** .pdf
**Title of data:** Additional figure 1
**Description of data:** Direct sequencing to rule out the occurrence of the nonsense-mediated mRNA decay mechanism. The *DNMT3A* exons 14-15 junction is shown, highlighting the two truncating variants.

**File format:** .pdf
**Title of data:** Additional figure 2
**Description of data:** Multiple Sequence Alignment (MSA) of DNMT3A C-terminal domains sampled from 6 eukaryotic model organisms and the 2 crystallized structures used to build the investigated mutants. The MSA was built using ClustalW implemented in JalView. The Zappo style was used to highlight conservation along the aligned sequences.

**File format:** .pdf
**Title of data:** Additional figure 3
**Description of data:** Multidimensional scaling (MDS) plot showing relations between technical replicates and samples. (R1: replicate 1; R2: replicate 2).
