# Supplementary figures and images for: Case report: biallelic DNMT3A mutations in acute myeloid leukemia
Source: Front Oncol. 2023 Jun 28;13:1205220. doi: 10.3389/fonc.2023.1205220 (PMC10336536; doi:10.3389/fonc.2023.1205220)

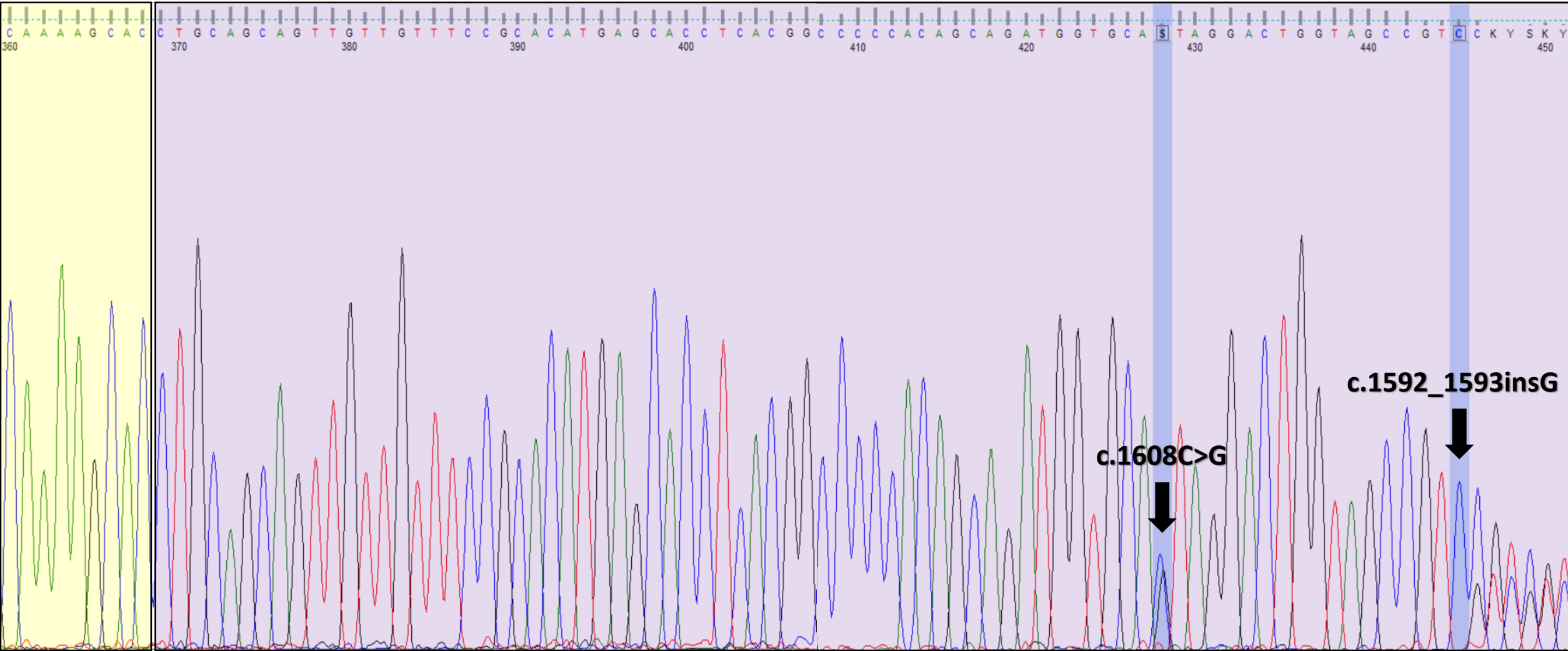

exon 15

exon 14

Supplement: Supplementary file 2 [file DataSheet_2.pdf]

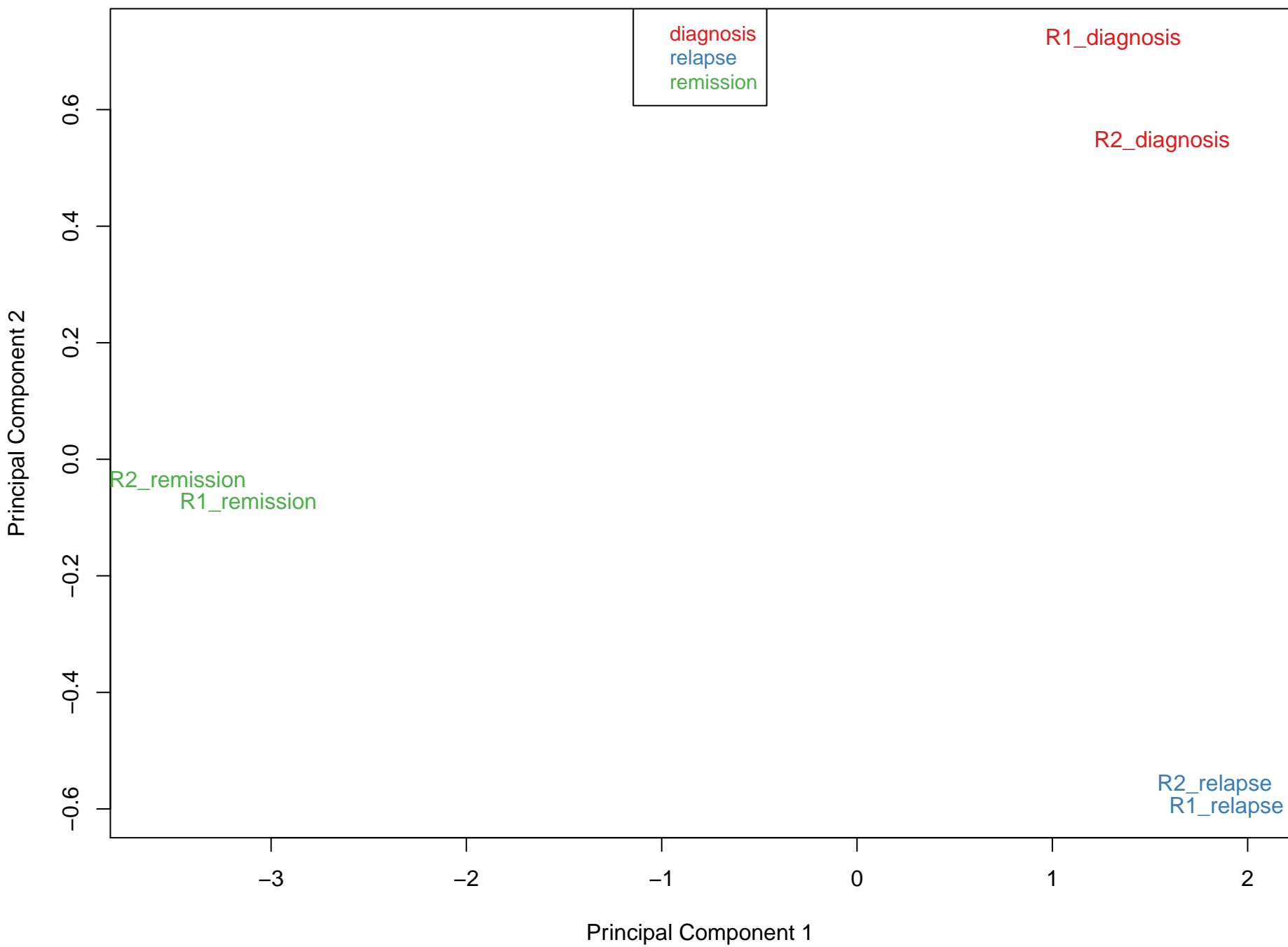

Supplement: Supplementary file 4 [file DataSheet_4.pdf]
