# Supplementary material for: Case report: biallelic DNMT3A mutations in acute myeloid leukemia
Source: Front Oncol. 2023 Jun 28;13:1205220. doi: 10.3389/fonc.2023.1205220 (PMC10336536; doi:10.3389/fonc.2023.1205220)
Supplement: Supplementary file 3 [file DataSheet_3.pdf]

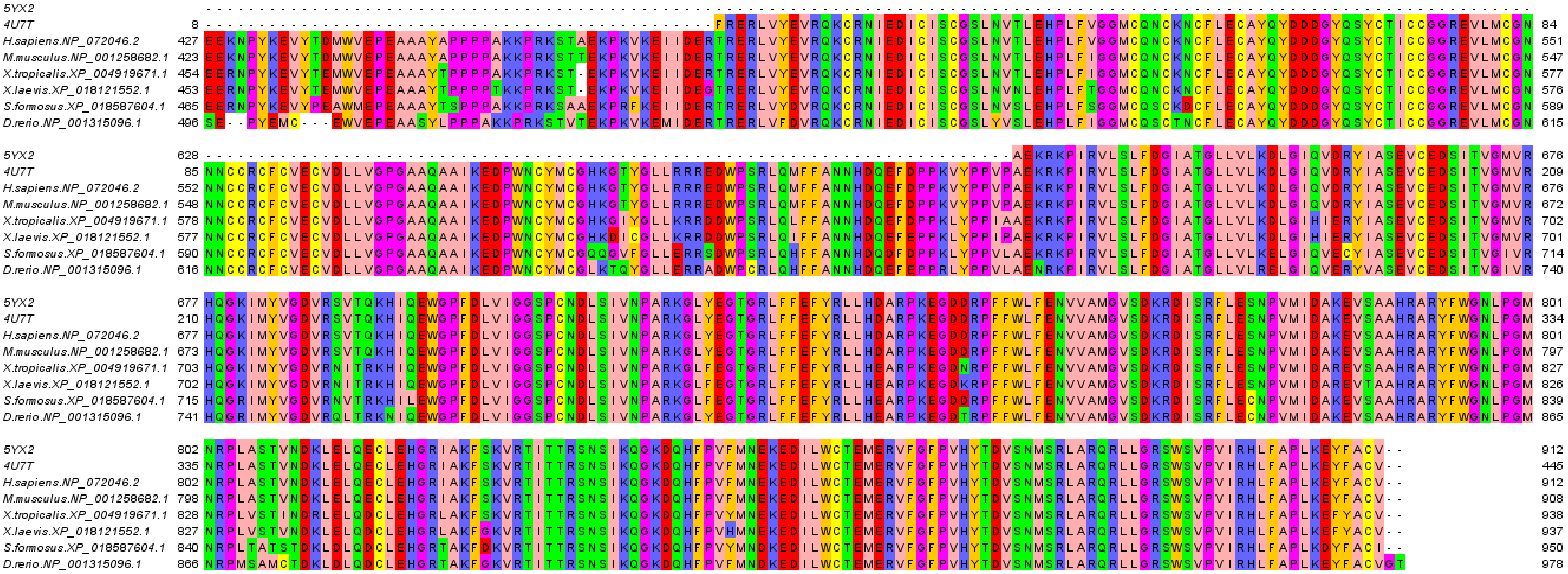
5YX2 ....
4U7T 8 ....FRERLVYEVRQKCRNIEDICISCGSLNVTLEHPLFVGGMCQNCKNCFLECAYQYDDDGYQSYCTICCGGREVLMCGN 84
H.sapiens.NP_072046.2 427 EEKNPYKEVYTDMWVEPEAAAYAPPPPAKKPRKSTAEKPKVKEIIDERTRERLVYEVRQKCRNIEDICISCGSLNVTLEHPLFVGGMCQNCKNCFLECAYQYDDDGYQSYCTICCGGREVLMCGN 551
M.musculus.NP_001258682.1 423 EEKNPYKEVYTDMWVEPEAAAYAPPPPAKKPRKSTTEKPKVKEIIDERTRERLVYEVRQKCRNIEDICISCGSLNVTLEHPLFIGGMCQNCKNCFLECAYQYDDDGYQSYCTICCGGREVLMCGN 547
X.tropicalis.XP_004919671.1 454 EEKNPYKEVYTEMWVEPEAAAYTPPPPAKKPRKST-EKPKVKEIIDERTRERLVYEVRQKCRNIEDICISCGSLNVTLEHPLFIGGMCQNCKNCFLECAYQYDDDGYQSYCTICCGGREVLMCGN 577
X.laevis.XP_018121552.1 453 EERNPYKEVYTEMWVEPEAAAYTPPPPTKKPRKST-EKPKVKEIIDEGTRERLVYEVRQKCRNIEDICISCGSLNVNLEHPLFTGGMCQNCKNCFLECAYQYDDDGYQSYCTICCGGREVLMCGN 576
S.formosus.XP_018587604.1 465 EERNPYKEVYPEAWMEPEAAAYTSPPPAKKPRKSAAEKPRFKEIIDERTRERLVYDVRQKCRNIEDICISCGSLNVSLEHPLFSGGMCQSCKDCFLECAYQYDDDGYQSYCTICCGGREVLMCGN 589
D.rerio.NP_001315096.1 496 SE--PYEMC---EWVEPEAASYLPPPAKKPRKSTVTEKPKVKEMIDERTRERLVFDVRQKCRNIEDICISCGSLYVSLEHPLFIGGMCQSCTNCFLECAYQYDDDGYQSYCTICCGGREVLMCGN 615
5YX2 628 ....AEKRKPIRVLSLFDGIATGLLVLKDLGIQVDRYIASEVCEDSITVGMVR 676
4U7T 85 NNCCRCFCVECVDLLVGPGAAQAAIKEDPWNCYMCGHKGTYGLLRRREDWPSRLQMFFANNHDQEFDPPKVYPPVPAEKRKPIRVLSLFDGIATGLLVLKDLGIQVDRYIASEVCEDSITVGMVR 209
H.sapiens.NP_072046.2 552 NNCCRCFCVECVDLLVGPGAAQAAIKEDPWNCYMCGHKGTYGLLRRREDWPSRLQMFFANNHDQEFDPPKVYPPVPAEKRKPIRVLSLFDGIATGLLVLKDLGIQVDRYIASEVCEDSITVGMVR 676
M.musculus.NP_001258682.1 548 NNCCRCFCVECVDLLVGPGAAQAAIKEDPWNCYMCGHKGTYGLLRRREDWPSRLQMFFANNHDQEFDPPKVYPPVPAEKRKPIRVLSLFDGIATGLLVLKDLGIQVDRYIASEVCEDSITVGMVR 672
X.tropicalis.XP_004919671.1 578 NNCCRCFCVECVDLLVGPGAAQAAIKEDPWNCYMCGHKGIYGLLKRRDDWPSRLQLFFANNHDQEFDPPKLYPPIAAEKRKPIRVLSLFDGIATGLLVLKDLGIHIERYIASEVCEDSITVGMVR 702
X.laevis.XP_018121552.1 577 NNCCRCFCVECVDLLVGPGAAQAAIKEDPWNCYMCGHKDICGLLKRRDDWPSRLQIFFANNHDQEFEPPKLYPPIPAEKRKPIRVLSLFDGIATGLLVLKDLGIHIERYIASEVCEDSITVGMVR 701
S.formosus.XP_018587604.1 590 NNCCRCFCVECVDLLVGPGAAQAAIKEDPWNCYMCGQQGVFGLLERRSDWPSRLQHFFANNHDQDFDPPKLYPPVLAEKRKPIRVLSLFDGIATGLLVLKELGIQVECYIASEVCEDSITVGIVR 714
D.rerio.NP_001315096.1 616 NNCCRCFCVECVDLLVGPGAAQAAIKEDPWNCYMCGLKTQYGLLERRADWPCRLQHFFANNHDQEFEPPRLYPPVLAENRKPIRVLSLFDGIATGLLVLRELGIQVERYVASEVCEDSITVGIVR 740
5YX2 677 HQGKIMYVGDVRSVTQKHIQEWGPFDLVIGGSPCNDLSIVNPARKGLYEGTGRLFFEFYRLLHDARPKEGDDRPFFWLFENVVAMGVSDKRDISRFLESNPVMIDAKEVSAAHRARYFWGNLPGM 801
4U7T 210 HQGKIMYVGDVRSVTQKHIQEWGPFDLVIGGSPCNDLSIVNPARKGLYEGTGRLFFEFYRLLHDARPKEGDDRPFFWLFENVVAMGVSDKRDISRFLESNPVMIDAKEVSAAHRARYFWGNLPGM 334
H.sapiens.NP_072046.2 677 HQGKIMYVGDVRSVTQKHIQEWGPFDLVIGGSPCNDLSIVNPARKGLYEGTGRLFFEFYRLLHDARPKEGDDRPFFWLFENVVAMGVSDKRDISRFLESNPVMIDAKEVSAAHRARYFWGNLPGM 801
M.musculus.NP_001258682.1 673 HQGKIMYVGDVRSVTQKHIQEWGPFDLVIGGSPCNDLSIVNPARKGLYEGTGRLFFEFYRLLHDARPKEGDDRPFFWLFENVVAMGVSDKRDISRFLESNPVMIDAKEVSAAHRARYFWGNLPGM 797
X.tropicalis.XP_004919671.1 703 HQGKIMYVGDVRNITRKHIQEWGPFDLVIGGSPCNDLSIVNPARKGLFEGTGRLFFEFYRLLHEARPKEGDNRPFFWLFENVVAMGVSDKRDISRFLESNPVMIDAREVSAAHRARYFWGNLPGM 827
X.laevis.XP_018121552.1 702 HQGKIMYVGDVRNITRKHIQEWGPFDLVIGGSPCNDLSIVNPARKGLFEGTGRLFFEFYRLLHEARPKEGDKRPFFWLFENVVAMGVSDKRDISRFLESNPVMIDAREVTAAHRARYFWGNLPGM 826
S.formosus.XP_018587604.1 715 HQGRIMYVGDVRNVTRKHILEWGPFDLVIGGSPCNDLSIVNPARKGLFEGTGRLFFEFYRLLHEARPKEGDDRPFFWLFENVVAMGVSDKRDISRFLECNPVMIDAKEVSAAHRARYFWGNLPGM 839
D.rerio.NP_001315096.1 741 HQGRIMYVGDVRQLTRKNIQEWGPFDLVIGGSPCNDLSIVNPARKGLYEGTGRLFFEFYRLLHEARPKEGDTRPFFWLFENVVAMGVSDKRDISRFLECNPVMIDAKEVSAAHRARYFWGNLPGM 865
5YX2 802 NRPLASTVNDKLELQECLEHGRIAKFSKVRTITTRSNSIKQGKDQHFPVFMNEKEDILWCTEMERVFGFPVHYTDVSNMSRLARQRLLGRSWSVPVIRHLFAPLKEYFACV-- 912
4U7T 335 NRPLASTVNDKLELQECLEHGRIAKFSKVRTITTRSNSIKQGKDQHFPVFMNEKEDILWCTEMERVFGFPVHYTDVSNMSRLARQRLLGRSWSVPVIRHLFAPLKEYFACV-- 445
H.sapiens.NP_072046.2 802 NRPLASTVNDKLELQECLEHGRIAKFSKVRTITTRSNSIKQGKDQHFPVFMNEKEDILWCTEMERVFGFPVHYTDVSNMSRLARQRLLGRSWSVPVIRHLFAPLKEYFACV-- 912
M.musculus.NP_001258682.1 798 NRPLASTVNDKLELQECLEHGRIAKFSKVRTITTRSNSIKQGKDQHFPVFMNEKEDILWCTEMERVFGFPVHYTDVSNMSRLARQRLLGRSWSVPVIRHLFAPLKEYFACV-- 908
X.tropicalis.XP_004919671.1 828 NRPLVSTINDRLELQDCLEHGRLAKFSKVRTITTRSNSIKQGKDQHFPVYMNEKEDILWCTEMERVFGFPVHYTDVSNMSRLARQRLLGRSWSVPVIRHLFAPLKEFYACV-- 938
X.laevis.XP_018121552.1 827 NRPLVSTVNDKLELQDCLEHGRLAKFGKVRTITTRSNSIKQGKDQHFPVHMNEKEDILWCTEMERVFGFPVHYTDVSNMSRLARQRLLGRSWSVPVIRHLFAPLKEFYACV-- 937
S.formosus.XP_018587604.1 840 NRPLTATSTDKLDLQDCLEHGRTAKFDKVRTITTRSNSIKQGKDQHFPVYMNDKEDILWCTEMERVFGFPVHYTDVSNMSRLARQRLLGRSWSVPVIRHLFAPLKDYFACI-- 950
D.rerio.NP_001315096.1 866 NRPMSAMCTDKLDLQDCLEHGRTAKFGKVRTITTRSNSIKQGKDQHFPVFMNDKEDILWCTEMERVFGFPVHYTDVSNMSRLARQRLLGRSWSVPVIRHLFAPLKEYFACVGT 978
